# Correlation between C-reactive Protein with Malondialdehyde in Systemic Lupus Erythematosus Patients

**DOI:** 10.1155/2020/8078412

**Published:** 2020-07-01

**Authors:** Nur Atik, S. Putri Pratiwi, Laniyati Hamijoyo

**Affiliations:** ^1^Department of Biomedical Sciences, Faculty of Medicine, Padjadjaran University, Bandung, Indonesia; ^2^Lupus Study Centre, Immunology Study Group, Padjadjaran University/Hasan Sadikin Hospital, Bandung, West Java, Indonesia; ^3^Undergraduate Program, Faculty of Medicine, Padjadjaran University, Bandung, Indonesia; ^4^Division of Rheumatology, Department of Internal Medicine, Faculty of Medicine, Padjadjaran University/Dr. Hasan Sadikin General Hospital Bandung, Indonesia

## Abstract

Systemic lupus erythematosus (SLE) is a systemic autoimmune disease characterized by an inflammatory process. One of the inflammation markers that can be measured is C-reactive protein (CRP). Another indicator of inflammation is malondialdehyde (MDA), though it is still uncommon to be analyzed in SLE patients. The study looked for the MDA value and found a correlation with CRP. A cross-sectional study design with a correlative analytical was performed. CRP level data was taken from Hasan Sadikin lupus registry data, and MDA levels were analyzed from a bioarchive patient's serum. We collected the patients' data who had CRP level from Hasan Sadikin lupus registry and analysed MDA levels from the serum sample. MDA levels were analyzed using an ELISA method. The data obtained were analyzed using the Pearson correlation and Eta correlation test. The study involved 78 data patients as subjects. It was found that the median of CRP and MDA was 0.85 mg/l and 153.10 ng/ml, respectively. These results indicate that the CRP levels in SLE patients are still within normal limits. Statistical analysis showed no correlation between CRP and MDA level (*r* = 0.2, *P* > 0.05). Additionally, the correlation between CRP and MDA with organ involvement, such as lupus nephritis (LN), lupus cutaneous (LC), and lupus musculoskeletal (LM), showed no correlation (*F*_h_ < *F*_t_). There is no correlation between CRP and MDA levels in SLE patients, and specific organ involvement of the disease does not affect the correlation.

## 1. Introduction

Systemic lupus erythematosus (SLE) is a systemic, chronic, and multisystem autoimmune disease that attacks various organs of the body caused by the deposition of immune complexes [1, 2]. This immune complex is caused by the failure of self-tolerance of B and T lymphocytes as well as the number of nuclear antigens originating from the fault in clearing dead cells which resulted from apoptosis, which causes a systemic inflammatory response and manifests according to the affected tissue or organ [1, 3–5]. Various inflammatory markers can be measured in SLE, one of which is C-reactive protein (CRP). A study states that CRP levels could present as normal or elevated in SLE patients as a sign of an inflammatory response [6]. CRP has been commonly used as a marker for inflammation, infection, tissue damage, malignancy, and autoimmune diseases [7].

CRP is a plasma protein synthesized by the liver, and their numbers increase during infections and inflammation. The presence of phagocytic cells that release IL-6 and IL-1 induces CRP production as a result of immune response [8]. Another indicator of oxidative stress and inflammation in SLE patients is malondialdehyde (MDA) [4]. Previous studies have shown a significant increase in MDA levels in SLE patients [4, 9]. MDA is a reactive aldehyde as the final result of a fat peroxidation process due to elevated levels of reactive oxygen species (ROS) [3, 4, 9]. ROS is generally formed as a result of cell metabolism in the body in small amounts. However, it causes harmful effects in excessive amount [10]. Other studies found that excess amount or production of ROS in SLE patients can be caused by several factors, such as an inflammatory response [3, 4, 9, 10]. These compounds then react with lipid membrane of the cells which become one of the targets prone to be damaged by ROS and produce hazardous compounds including MDA [4, 10].

The correlation between inflammatory conditions with the oxidation process is still poorly understood, specifically a relationship between the level of inflammation characterized by CRP levels and oxidative stress characterized by MDA levels in SLE patients. Previous studies have found that increased ROS correlates with CRP in lupus nephritis patients, and there is a correlation between inflammatory conditions that trigger oxidation in SLE patients [3]. Thus, we analyzed the CRP and MDA levels from the SLE patients to know the correlation for both inflammatory markers. This research is expected to be a source of data on CRP and MDA levels, especially MDA that are not included in the routine examination in SLE patients. It is also expected to find information about the correlation between CRP and MDA in SLE patients.

## 2. Materials and Methods

### 2.1. The Patients' Data Collection

The cross-sectional analytic study using clinical, CRP data and serum bioarchived samples from Hasan Sadikin Lupus Registry research, whereas CRP levels were taken from Hasan Sadikin Lupus Registry data [11]. We collected 78 patients' data that have been included in the inclusion criteria. The patients' data were registered in the Rheumatology Division of the Department of Internal Medicine Hasan Sadikin Hospital Bandung from January 1, 2014, to December 31, 2018, period. The exclusion criteria consist of patients with incomplete lupus data registry, and the serum samples were damaged. The minimum sample size needed for this research is 62, which is determined by the sample size formula for correlative analytics research.

This study is following the WMA Declaration of Helsinki on ethical principles for medical research, and the protocol was approved by the Research Ethics Committee of Padjadjaran University (685/UN6.KEP/EC/2019).

### 2.2. ELISA Procedure

An ELISA competitive method was used for MDA level testing in this study, conducted at the Clinical Pathology Laboratory of Hasan Sadikin Hospital Bandung. 78 serum samples from the subject bioarchive were proceeded to MDA analysis. All the sera were matched with the CRP value prior the MDA analysis. The serum samples had previously been frozen at -80°C and then thawed by placing the tube to room temperature (18-25°C) for 2 hours.

MDA was measured using an ELISA Kit Elabscience (according to the manufacturer's instructions) [12]. Briefly, 50 *μ*L of serum were tested in each well by adding 50 *μ*L of biotinylated detection antibody. It was followed by incubation for 45 minutes at 37°C. The reaction was washed three times. Add 100 *μ*L of HRP conjugate, incubate for 30 minutes at 37°C, and then wash five times. Add 90 *μ*L of substrate reagent then incubate for 15 minutes at 37°C. Add 50 *μ*L stop solution and determine the optical density (OD) value at 450 nm; then calculate the results. The MDA detection limit with the Competitive ELISA Kit method is 31.25-2000 ng/mL.

### 2.3. Statistical Analysis

The data collected were analysed using IBM SPSS Statistics version 23.0 statistical software. The analysis of data correlation between CRP and MDA was done using the Pearson correlation coefficient. The correlation between CRP and MDA levels with organ involvement was analysed using the Eta correlation coefficient test.

## 3. Results

### 3.1. SLE Patient Characteristics

There were 78 CRP patient data obtained, which were connected with patients' serum who had met the criteria. Data characteristics of the subjects of this study showed as many as 77 (98.72%) patients in this study were female. The average age of SLE patients in this study was 39 years. The duration or length of time the subject had is a median of 4.5 years. Most of the subjects work as a housewife (58.97%). There were 43 (55.13%) patients with kidney disorders involved in this study, 63 patients with skin disorders (80.77%), and 62 patients with disorders of muscles and bones (79.49%). The disease activity of SLE patients measured by SLEDAI-2 k showed that 47 patients (60.26%) were in an inactive immune system (Table 1).

### 3.2. Correlation between CRP and MDA

CRP and MDA levels of SLE patients in this study were found to have a median of 0.85 mg/L and 153.10 ng/mL, respectively. The median of CRP results showed no increase of CRP levels in the subjects. The lowest-highest value of each variable was 0.05-27.3 mg/L and 50.88-693.16 ng/mL. The analysis test showed the *r* value of the correlation coefficient between the two variables was 0.2 with a *P* value > 0.05, so there was no correlation between CRP and MDA in SLE patients analyzed by the Pearson correlation method (Table 2).

### 3.3. Correlation between CRP and MDA with Organ Involvement

A study in Brazil found that increased ROS correlated with CRP in lupus nephritis patients [3]. Increased free radicals were also found in proliferative lupus nephritis patients compared with lupus patients who did not have kidney abnormalities or lupus patients without nephritis. In lupus cutaneous patients, an increase in free radicals is in accordance with the activity of SLE disease, especially after exposure to ultraviolet light [13]. The occurrence of an imbalance of ROS with antioxidants in the body is what causes oxidative stress [14]. The excess production of ROS in the body is also considered to have a relationship with inflammatory conditions [15, 16]. Therefore, to find out whether each organ involvement influences the concentration of CRP or MDA levels, the Eta correlation coefficient test was performed (Table 3).

Based on the results of the analysis, the coefficient of *η* = 0.127 was obtained in the correlation of CRP with lupus nephritis, *η* = 0.083 with lupus cutaneous, and *η* = 0.066 with lupus musculoskeletal. Furthermore, the coefficient value *η* = 0.026 was obtained in the correlation of MDA with lupus nephritis, *η* = 0.012 with lupus cutaneous, and *η* = 0.114 with lupus musculoskeletal. It means that CRP and MDA values with organ involvement in lupus patients in this study had a very low or weak correlation. Furthermore, we proceed the data with the *F* test to know the correlation between CRP and MDA to the specific organs that affected in the SLE patients. We found that all results showed *F*_count_ < *F*_table_. It means that there is no significant correlation between CRP and MDA values with organ involvement in lupus patients (Table 3).

## 4. Discussion

This study shows that the prevalence of female SLE patients (98.72%) is greater than that of men (1.28%). These results are consistent with a previous study, which states that SLE attacks more women [11, 17]. The average age of the subjects is 39 years. This is also in accordance with the same previous study that the highest numbers of SLE disease occurred in the age range of 30-49 years and 50-64 years [17]. The duration of lupus illness has a median of 4.5 years with a duration range of 1-18 years. These results are different from other studies which shows a range of duration of SLE disease ranging from 0 to 15 years with a median of 9 years [3]. Previous studies in West Java, Indonesia, had an average duration of illness of 76.5 months [11]. The involvement of organs in this study mostly attacks the skins (80.77%) then followed by musculoskeletal disorders (79.49%) and the kidneys (55.13%). This study is slightly different from other studies which state that 85% of the complications are involved in musculoskeletal and arthritis, 75% in the skin and mucosa, and 30% in kidney disorders [18].

A characteristic of SLE is the presence of an immune complex that activates the endosomal toll-like receptor (TLR) and triggers the inflammatory process. Various markers of inflammation can be measured in SLE patients, such as CRP [6]. CRP levels can be used as an indicator of low-grade inflammation in infections and chronic diseases [19–21]. The median value of CRP in this study is 0.85 mg/L, known as the normal level [20]. A CRP level can be normal or elevated in SLE patients as a sign of an inflammatory response [6]. Other studies have shown that an average CRP level > 10 mg/L in both SLE patients who were active, inactive, exacerbation, infected, or not [22]. High CRP levels > 10 mg/L can indicate active or flare SLE patients [23, 24]. A previous study showed an average CRP value of 323.5 mg/dL with a median of 48 mg/dL, and others showed 3.41 mg/L and 1.57 mg/L [25–27]. Factors that can affect the diversity of high or low CRP levels include psychosocial stress, low education, antimalaria drugs, infections such as serositis, Body Mass Index (BMI), and ethnicity. It is known that the disease activity is correlated with the inflammatory response [28, 29]. We showed that the majority of patients' serum samples were taken in an inactive stage of the immune system. It caused the CRP levels of patients in this study to be within normal limits.

In addition, most of the subjects in this study work as housewives. Based on the WHO's “Global Strategy on Diet, Physical Activity, and Health,” housewife activities are categorized as moderate physical activity. Moderate-intensity physical activity can also reduce the inflammatory response by suppressing expressions of TLR 4 on monocytes [27]. The mechanism of physical activity affecting TLR expression is yet to be clearly known, but there is a possible role of anti-inflammatory cytokines and heat shock protein (HSP) involvement [30, 31]. Binding of HSP with TLR 4 can cause an inflammatory response. A study proves that physical activity can cause downregulation of HSP [32]. Another important factor influencing the inflammatory response is psychosocial stress. It causes the secretion of proinflammatory cytokines (IL-1 and IL-6) and stress hormones (corticosteroids and catecholamines) thereby triggering inflammatory processes and CRP [33]. The involvement of gene polymorphisms in CRP also affects the course of the disease and causes low CRP levels during flare conditions in SLE patients [23].

In SLE patients, an increase of ROS affects the severity of the disease [3, 34, 35]. The ROS causes oxidative stress conditions, reactive autoantibodies, autoantigen production, and immune cell disorders [3, 13]. The imbalance of ROS levels with body antioxidants causes oxidative stress conditions and triggers fat peroxidation reactions and produces the MDA [3, 4, 10, 16]. In this study, the median of MDA value in patients was 153.10 ng/mL. Other research in Iran shows a median MDA level of 1.84 *μ*mol/L and states that an increasing MDA level as an indicator of inflammation and oxidative stress is related to the presence of lupus disease [4]. The MDA levels may increase due to mitochondrial dysfunction in SLE patients, external exposure such as cigarettes and ultraviolet (UV) rays, and low levels of glutathione as an antioxidant in lupus patients. Previous studies found that there is a correlation between inflammatory conditions that trigger the oxidation process in SLE patients [3]. However, our results showed that there was no correlation between CRP and MDA in SLE patients in this study (*r* = 0.2, *P* > 0.05).

Additionally, we tested the correlation between CRP and MDA levels in patients with lupus nephritis, lupus musculoskeletal, and lupus cutaneous. Previous studies suggest that increased ROS correlates with CRP in lupus nephritis patients [3]. Other studies state that oxidative stress triggers inflammatory processes and the incidence of lupus nephritis [36]. Increased free radicals are found in proliferative lupus nephritis patients compared with lupus patients without kidney disorders. Besides, an increase in free radicals also occurs in the skin organs of lupus cutaneous patients, which happens in accordance with SLE, especially after exposure to ultraviolet rays [13]. However, among the three analysis results, we did not find any correlation between both CRP and MDA levels with organ involvement (*F*_count_ < *F*_table_). CRP as a marker of inflammation can still be found within normal limits in SLE patients. As an additional, there is no correlation between CRP and MDA in SLE patients, and also, there is no correlation between CRP and MDA levels with organ involvement.

This first study regarding CRP and MDA correlation in Indonesia SLE patients has several limitations. The recent study only focuses on the status of CRP and MDA without observing deeply for the infection status and the treatment history as well. The limited number of subjects could also be a limitation for this study.

## 5. Conclusion

CRP as a marker of inflammation can still be found within normal limits in SLE patients. As an additional, there is no correlation between CRP and MDA in SLE patients, and also, there is no correlation between CRP and MDA levels with organ involvement.

## Figures and Tables

**Table 1:** SLE patient characteristics.

Variable	SLE patients (*n* = 78)
Gender:	
(1) Male	1 (1.28%)
(2) Female	77 (98.72%)
Age^b^	39 (9.55)
Duration or length of disease^c^ (counted from the date of diagnosis to the date the blood was drawn)	4.5 (1-18)
Jobs^a^	
(i) Housewives	46 (58.97%)
(ii) Employees	8 (10.26%)
(iii) Teachers	7 (8.97%)
(iv) Private employees	5 (6.41%)
(v) Students	2 (2.56%)
(vi) Government employees	1 (1.28%)
(vii) Professional	1 (1.28%)
(viii) Others	2 (2.56%)
(ix) Unemployed	1 (1.28%)
(x) No data	5 (6.41%)
Organ involvement^a^	
(i) Lupus nephritis	43 (55.13%)
(ii) Lupus cutaneous	63 (80.77%)
(iii) Lupus musculoskeletal	62 (79.49%)
SLEDAI-2k^a^	
(i) <4	47 (60.26%)
(ii) ≥4	31 (39.74%)

^a^Amount (percentage). ^b^Mean (average). ^c^Median (minimum-maximum).

**Table 2:** Correlation coefficient of CRP and MDA.

Variables	SLE patients (*n* = 78)	*r* (*P*)
CRP^a^	0.85 (0.05-27.30)	0.2 (0.08)
MDA^a^	153.10 (50.88-693.16)	

**Table 3:** Coefficient correlation of CRP or MDA with organ involvement.

Variable	*η*	*F*_count_	*F*_table_	Interpretation
CRP and LN correlation	0.127	1.246	3.97	*F*_h_ < *F*_t_
CRP and LC correlation	0.083	0.527	3.97	*F*_h_ < *F*_t_
CRP and LM correlation	0.066	0.333	3.97	*F*_h_ < *F*_t_
MDA and LN correlation	0.026	0.051	3.97	*F*_h_ < *F*_t_
MDA and LC correlation	0.012	0.011	3.97	*F*_h_ < *F*_t_
MDA and LM correlation	0.114	1.001	3.97	*F*_h_ < *F*_t_

LN: lupus nephritis; LC: lupus cutaneous; LM: lupus musculoskeletal.

## Data Availability

The data used to support the findings of this study are available from the corresponding author upon request.

## Abbreviations

SLE: Systemic lupus erythematosus
CRP: C-reactive protein
MDA: Malondialdehyde
ELISA: Enzyme-Linked Immunosorbent Assay
ROS: Reactive oxygen species
HRP: Horseradish peroxidase
OD: Optical density
SLEDAI: Systemic Lupus Erythematosus Disease Activity Index
LN: Lupus nephritis
LC: Lupus cutaneous
LM: Lupus musculoskeletal
TLR: Toll-like receptor
HSP: Heat shock protein.
